# Construction of a tri-chromatic reporter cell line for the rapid and simple screening of splice-switching oligonucleotides targeting DMD exon 51 using high content screening

**DOI:** 10.1371/journal.pone.0197373

**Published:** 2018-05-16

**Authors:** Takenori Shimo, Keisuke Tachibana, Satoshi Obika

**Affiliations:** Graduate School of Pharmaceutical Sciences, Osaka University, Suita, Osaka, Japan; University of Minnesota Medical Center, UNITED STATES

## Abstract

Splice-switching oligonucleotides (SSOs) that can modulate RNA splicing are used for the treatment of many genetic disorders. To enhance the efficacy of modulating splicing, it is important to optimize SSOs with regard to target sites, GC content, melting temperature (*T*_m_ value), chemistries, and lengths. Thus, *in vitro* assay systems that allow for the rapid and simple screening of SSOs are essential for optimizing SSO design. In this study, we established a novel tri-chromatic reporter cell line for SSO screening. This reporter cell line is designed to express three different fluorescent proteins (blue, green, and red) and was employed for high content screening (HCS, also known as high content analysis; HCA) for the evaluation of SSO-induced exon skipping by analyzing the expression levels of fluorescent proteins. The blue fluorescent protein is stably expressed throughout the cell and is useful for data normalization using cell numbers. Furthermore, both the green and red fluorescent proteins were used for monitoring the splicing patterns of target genes. Indeed, we demonstrated that this novel reporter cell line involving HCS leads to a more rapid and simple approach for the evaluation of exon skipping than widely used methods, such as RT-PCR, western blotting, and quantitative RT-PCR. Additionally, a brief screening of Locked nucleic acids (LNA)-based SSOs targeting exon 51 in *DMD* was performed using the reporter cell line. The LNA-based SSO cocktail shows high exon 51 skipping in a dose-dependent manner. Furthermore, the LNA-based SSO cocktails display high exon 51 skipping activities on endogenous *DMD* mRNA in human rhabdomyosarcoma cells.

## Introduction

Antisense-mediated splicing modulation is an attractive therapeutic approach for many genetic disorders involving RNA mis-splicing [1]. A previous study revealed that over 60% of point mutations result in splicing errors [2]. Moreover, in 2016, the US Food and Drug Administration (FDA) approved two splice-switching oligonucleotides (SSOs): eteplirsen for the Duchenne muscular dystrophy (DMD) and nusinersen for spinal muscular atrophy (SMA) [3]. Thus, SSOs represent excellent candidates for the further development of medical therapies for genetic disorders.

Locked nucleic acids (LNA), also known as 2′-*O*,4′-*C*-methylene-bridged nucleic acids (2′,4′-BNA), represent well-known modified nucleic acids synthesized by the Wengel and our group respectively [4, 5]. Many studies regarding LNA-based SSOs have been reported by us and other groups [6–12]. Previously, we have screened and optimized LNA-based SSOs targeting exon 58 in *DMD*. We demonstrated that LNA-based SSOs show higher exon skipping activity than SSOs partially containing 2′-*O*-methyl RNA, at least for the skipping of exon 58 in *DMD*. Furthermore, *Le et al*. reported that short LNA-modified 2′-*O*-methyl SSOs (16- to 14-mer) show a higher exon skipping activity than the corresponding 2′-*O*-methyl RNA based SSO in *H-2K*^*b*^-tsA58 *mdx* mouse myotubes *in vitro* [13]. Moreover, *Touznik et al*. recently reported that LNA-based SSOs targeting the *SMN2* gene exhibit an increased splicing modulatory effect [14].

To develop novel antisense-based drugs, we and other groups have previously reported that the optimization of SSO design e.g., target sites, guanine-cytosine (GC) content, melting temperature (*T*_m_ value), chemistries and lengths should represent key elements [12, 15, 16]. Therefore, *in vitro* screening systems for the rapid and simple evaluation of exon skipping activity are important because of the need to design and compare many candidate SSOs. Until now, many studies have focused on reporter systems for the rapid screening of pre-mRNA splicing [17–25]. Amongst them, *Orengo et al*. have previously suggested an excellent strategy: a bi-chromatic reporter system that detects splicing patterns [20]. In their reporter system, two different fluorescent proteins are expressed based on exon skipping. Because this represents a simple strategy for monitoring splicing patterns, it has been applied for investigating the splicing patterns of many genes [26–30]. In these bi-chromatic reporter systems, the ratio of two fluorescent proteins is used for the evaluation of splicing patterns. However, for assessing the exon skipping activity of SSOs, it is also important to normalize the data by using the cell number because aberrant SSO-induced splicing might affect the expression of the fluorescent proteins.

To perform multidimensional screening, a novel approach known as high content screening (HCS, also known as HCA: high content analysis), has recently been developed. HCS is very advantageous for detecting fluorescence intensity more precisely and rapidly than conventional methods such as fluorescence microscopy and fluorescence plate readers [31]. Concurrently, it is possible to count the number of cells during HCS. Therefore, HCS has often been applied in various biological contexts [32, 33], and is employed for the drug screening of small molecules, especially in industry [34]. However, to our knowledge, there are few reports regarding the use of HCS for evaluating the efficiency of exon skipping. Therefore, a novel assay system for evaluation of SSOs using HCS is required.

In the present study, we have established a novel tri-chromatic reporter cell line for the rapid and simple evaluation of the skipping efficiency of *DMD* exon 51 using SSOs and HCS. Moreover, we performed a brief screening of LNA-based SSOs cocktails using our reporter cell line and found that a 3-mix cocktail of LNA-based SSOs displays a higher exon skipping activity. Additionally, we demonstrate that the 3-mix LNA-based SSO cocktails promote the skipping of exon 51 in *DMD* at the endogenous *DMD* locus in the human rhabdomyosarcoma cell line.

## Materials and methods

### Synthesis of oligonucleotides

All SSOs used in this study are shown in S1 Table. LNA was partially incorporated into the SSO sequences, in which the phosphodiester linkages were completely replaced by phosphorothioate linkages. LNA-based SSOs and PRO-051 were designed to have sequences complementary to the human dystrophin gene and were synthesized and purified by GeneDesign Inc. (Osaka, Japan). AVI-4658 was purchased from Gene Tools (Philomath, OR).

### Plasmid construction

We used standard cloning techniques reported in a previous study for the construction of the reporter plasmid [12, 20]. The nuclear localization signal (NLS) was constructed by annealing the 5′-CTGCCCCAAAAAAAAAACGCAAAGTGGAGGACCCATTCGAAGTG-3′ forward oligonucleotide (the underlined sequence corresponds to the BstBI site) and the 5′-GATCCACTTCGAATGGGTCCTCCACTTTGCGTTTTTTTTTTGGGGCAGGTAC-3′ reverse oligonucleotide. The annealed oligonucleotide was cloned into the KpnI-BamHI sites in pcDNA5/FRT-FLAG-DsRed-EGFP [12] (designated as pcDNA5/FRT-FLAG-NLS-DsRed-EGFP-2).

The human *DMD* minigene containing *DMD* exons 50–52 was isolated as described below. Because both intron 50 and 51 sequences are too long to insert in a plasmid, the *DMD* minigene was designed to have short sequences flanking intron 50 (removing the sequence from position +161 to +45,612) and 51 (removing the sequence from position +151 to +44,056). At first, we amplified three DNA fragments using different primer sets. DNA fragment-1 that contains both exon 50 and the short 5′ sequence of intron 50 and was amplified with forward primer-1 (5′-AATTCGAATTAAAGAGGAAGTTAGAAGAT-3′, where the underlined sequence corresponds to the BstBI site) and reverse primer-1 (5′-AATGTGGATAAAGGAATGTACTCTAAGACT-3′). DNA fragment-2 contains exon 51 and was amplified with forward primer-2 (5′-TACATTCCTTTATCCACATTGTTAGAAAAA-3′) and reverse primer-2 (5′-CAATTTAACATTAGGCTGAATAGTGAGAGT-3′). DNA fragment-3 contains exon 52 and was amplified with forward primer-3 (5′-TTCAGCCTAATGTTAAATTGTTTTCTATAA-3′), and reverse primer-3 (5′-ACACCGGTAACCTCTTGATTGCTGGTCTTGTTTT-3′, where the underlined sequence corresponds to the AgeI site). Next, we mixed DNA fragments-1 and -2 as templates, and amplified DNA fragment-4 using forward primer-1 and reverse primer-2. Then, we mixed DNA fragment-4 and fragment-3 as templates, and amplified the *DMD* minigene using forward primer-1 and reverse primer-3. The amplified *DMD* minigene was digested with the BstBI and AgeI restriction enzymes and cloned into the BstBI-AgeI sites of pcDNA5/FRT-FLAG-NLS-DsRed-EGFP-2 (designated as pcDNA5/FRT-FLAG-NLS-DMD-Exon50-51-52-DsRed-EGFP).

The EGFP fragment was amplified from the pcDNA5/FRT-FLAG-NLS-DsRed-EGFP [12] by PCR using forward primer-4 (5′-GTTACCGGTGTGGTGAGCAAGGGCGAGGAGCT-3′, where the underlined sequence corresponds to the AgeI site) and the 5′-CTGCGTTAACTGTTACTTGTACAGCTCGTCCA-3′ reverse primer (the underlined sequence corresponds to the HpaI site). The Halotag fragment was amplified from pFN21AB901 (Kazusa DNA Research Institute) by PCR using the 5′-GTAACAGTTAACGCAGAAATCGGTACTGGCTT-3′ forward primer (the underlined sequence corresponds to the HpaI site) and reverse primer-4 (5′-AACGGGCCCTCAGCCGGAAATCTCGAGCGTCG-3′, where the underlined sequence corresponds to the ApaI site). These two fragments were mixed and underwent a second round of PCR using forward primer-4 and reverse primer-4. The DNA fragment encoding DsRed and EGFP in pcDNA5/FRT-FLAG-NLS-DMD-exon50-51-52-DsRed-EGFP were removed using the AgeI and ApaI restriction enzymes. The resulting PCR product, that was digested using the AgeI and ApaI restriction enzymes, was cloned into the AgeI-ApaI sites (termed pcDNA5/FRT-Flag-NLS-DMD-exon50-51-52-EGFP-Halotag).

The TagRFP fragment was amplified by PCR using the 5′-TGACAGTTAACGTGTCTAAGGGCGAAGAGCT-3′ forward primer (the underlined sequence corresponds to the HpaI site) and the 5′-AACGGGCCCTCAATTAAGTTTGTGCCCCA-3′ reverse primer (the underlined sequence corresponds to the ApaI site). The gene encoding the Halotag in pcDNA5/FRT-Flag-NLS-DMD-exon50-51-52-EGFP-Halotag was removed using the HpaI and ApaI restriction enzymes, and the resulting TagRFP fragment, digested with HpaI and ApaI, was cloned into the HpaI-ApaI sites (named pcDNA5/FRT-Flag-NLS-DMD-exon50-51-52-EGFP-TagRFP). We used this plasmid as the reporter minigene for *DMD* exon 51 skipping. All constructs were verified by DNA sequencing.

### Cell culture

Flp-In CHO cells (Invitrogen, Waltham, MA) were cultured in Ham’s F12 medium containing 10% fetal bovine serum (FBS) (Biowest, Nuaillé, France), 1x Antibiotic-Antimycotic Solution for cell culture (Sigma-Aldrich, St. Louis, MO), 100 μg/mL Zeocin (Thermo Fisher Scientific, Waltham, MA) and maintained in a 5% CO_2_ incubator at 37 °C. The human rhabdomyosarcoma (RD) cell line (JCRB cell bank) was cultured in DMEM containing 10% FBS and 1x Antibiotic-Antimycotic Solution for cell culture.

### Generation of the reporter cell line

The Flp-In CHO cell line was used for the establishment of the reporter cell line. At first, we established a stable cell line that expresses TagBFP proteins. The cells were seeded into a 6-cm dish 24 h before plasmid DNA transfection. The pTagBFP-N vector (Evrogen, Moscow, Russia) was linearized using the AflIII restriction enzyme (New England Biolabs, Ipswich, MA). The linearized pTagBFP-N vector was transfected into Flp-In CHO cells using Lipofectamine 2000 (Thermo Fisher Scientific). Cells stably expressing the vector were selected using 150 μg/mL G-418, and designated as Flp-In CHO-BFP.

Next, we established the reporter cell line that expresses both TagBFP and the reporter gene. The Flp-In CHO-BFP cells were seeded into a 6-cm dish 24 h before transfection. The reporter plasmid, pcDNA5/FRT-Flag-NLS-DMD-exon50-51-52-EGFP-TagRFP, and pOG44 (the flp recombinase expression plasmid) were both transfected into Flp-In CHO-BFP cells. The stable reporter cell line was selected using 800 μg/mL Hygromycin B, and designated as Flp-In CHO-BFP-DMD-exon51-GFP-RFP. This reporter cell line was cultured in Ham’s F12 medium containing 10% FBS, 1x Antibiotic-Antimycotic Solution for cell culture, 200 μg/mL Hygromycin B, and 200 μg/mL G-418.

### SSO transfection into the reporter cell line

For SSO transfection, the established reporter cell line was seeded one day before transfection at a density of 12,500 cells/well in 96-well black plates (Corning, Corning, NY) or 80,000 cells/well in 24-well black plates (Greiner Bio-One, Kremsmuenster, Austria). After 24 h, the cells were transfected with SSOs using Lipofectamine 2000, according to the manufacturer’s protocols, and grown in DMEM containing 10% FBS and 1x Antibiotic-Antimycotic solution. Twenty-four hours after transfection, cells were used for various assays.

### SSO transfection into the RD cell line

Before SSO transfection, RD cells were seeded at a density of 80,000 cells/well in 24-well plates (Iwaki Co, Tokyo, Japan). The next day, the medium was exchanged with DMEM containing 10% FBS, 1x Antibiotic-Antimycotic solution and 100 nM 12-*O*-Tetradecanoylphorbol-13-Acetate (TPA) and the cells were incubated for six days to induce differentiation [35, 36]. The differentiated RD cells were transfected with 500 nM SSOs using the Lipofectamine 2000 reagent, according to the manufacturer’s protocols, and grown in DMEM containing 10% FBS, 1x Antibiotic-Antimycotic solution and 100 nM TPA. Only AVI-4658, a phosphorodiamidate morpholino oligomer (PMO)-based SSO, was transfected at 10 μM with Endo-porter (Gene Tools, Philomath, OR) according to the manufacturer’s protocols under the same conditions as above. Twenty-four hours after transfection, the cells were harvested and used for further experiments.

### HCS

ToxInsight (Thermo Fisher Scientific) was used for HCS, for the acquisition of fluorescence images of the reporter cell line in 96-well or 24-well black plates. The intensity of each fluorescent protein was measured using the corresponding LED-based illumination (386, 465, or 584 nm). The captured fluorescence images were analyzed using the Thermo Scientific Cellomics Spot Detector V4 program (Thermo Fisher Scientific). We used the MEAN_ObjectSpotTotalInenCh3 value for the evaluation of the fluorescence intensity for the TagRFP protein. Using this value, only cells that stably express TagBFP proteins are detected as objects. For each object, the TagRFP protein expression in each unit area was summed as the spot intensities for TagRFP. Next, all spot intensities were summed as total intensities. Finally, the total intensity of TagRFP proteins was normalized using the count of all objects in the respective well.

### Fluorescence microscopy

After performing HCS, the reporter cells on 96-well or 24-well black culture plates were analyzed using a BZ-8000 microscope (Keyence, Osaka, Japan). Phase contrast and fluorescence images were obtained at a magnification of 20x. Fluorescence images were collected using the (Ex/Em = 360/460 nm), (Ex/Em = 470/525 nm), and (Ex/Em = 545/605 nm) filters (Keyence) for TagBFP, EGFP, and TagRFP, respectively.

### RNA isolation and cDNA synthesis

Twenty-four hours after transfection, total RNA samples were isolated using the QuickGene 800 (Kurabo, Osaka, Japan) and QuickGene RNA cultured cell kit S (Kurabo) according to the manufacturer’s instructions. The reverse-transcription of 90 ng (from the reporter cell line) or 150 ng (from the RD cell line) of total RNA was performed using the Rever Tra Ace qPCR RT Master Mix (Toyobo, Osaka, Japan) according to the manufacturer’s instructions.

### RT-PCR analysis

A 1:60 (from the reporter cell line) or 1:1 (from the RD cell line) dilution of cDNA was used as a template for individual PCR reactions using specific primer sets (S2 and S3 Tables), that were designed using the Primer blast program [37]. For cDNA obtained from the RD cell line, nested PCR was performed. PCR was conducted using the KOD FX Neo DNA polymerase (Toyobo, Tokyo, Japan), and the PCR products were analyzed on a 2% agarose gel stained with ethidium bromide, with specific bands purified for sequence analysis. The intensity of each band was quantified using the ImageJ software (National Institutes of Health; freeware from http://rsb.info.nih.gov/ij/). Glyceraldehyde-3-phosphate dehydrogenases (*homo sapiens GAPDH* and *Cricetulus griseus GAPDH*) were used as internal controls. The exon skipping percentage was calculated as the amount of skipped *DMD* exon 50 transcript relative to the total amount of skipped *DMD* exon 50 plus full-length transcripts (in which exon 50 is included) [12].

### Quantitative RT-PCR analysis

A 1:60 cDNA dilution was used as a template for individual PCR reactions using specific primer sets (S4 Table), that were designed using the Primer blast program [37]. Quantitative RT-PCR analyses were performed using the SYBRGreen Real-time PCR Master Mix-Plus (Toyobo) and a StepOnePlus device (Thermo Fisher Scientific) according to manufacturer’s protocols; however, the annealing time was reduced to 15 sec and the annealing temperature was set at 65 °C. *Cricetulus griseus GAPDH* was used for normalizing the data. The amplification specificity of the PCR products was investigated on a 5% agarose gel stained with ethidium bromide.

### Western blot analysis

To confirm the expression of each fluorescent and Flag-tagged protein, western blotting was performed. Twenty-four hours after SSO transfection, total proteins were extracted using the T-per Tissue Protein Extract Reagent (Thermo Fisher Scientific) and the Halt protease inhibitor cocktail. Forty to 400 ng of total proteins were loaded onto NuPAGE 4–12% Bis-Tris Protein Gels (Thermo Fisher Scientific), following the manufacturer’s protocols. The gels were loaded onto a XCell Sure Lock Mini Cell system (Thermo Fisher Scientific) and run in NuPAGE MES SDS Running Buffer (Thermo Fisher Scientific) at 200 mA for 40 min. The gels were transferred to PVDF membranes by semidry blotting at 20 V for 60 min [38]. A mouse monoclonal antibody against RFP (#ab125244, Abcam, Cambridge, MA), a mouse monoclonal antibody against EGFP (#632569, Clontech, Mountain View, CA), a rabbit polyclonal antibody against FLAG-conjugated proteins (#600-401-383, Rockland Immunochemicals, Gilbertsville, PA) and a rabbit monoclonal antibody against β-actin (Cell Signaling Technology, Danvers, MA) were used as primary antibodies. The primary antibodies were diluted in Can Get Signal Immunoreaction Enhancer Solution 1 (Toyobo). The PVDF membranes were incubated with the primary antibodies overnight at 4 °C. Horseradish peroxidase-conjugated anti-rabbit or anti-mouse goat immunoglobulins (R&D Systems, Minneapolis, MN) were used as secondary antibodies. The secondary antibodies were diluted in Can Get Signal Immunoreaction Enhancer Solution 2 (Toyobo). The Chemi-Lumi One Super kit (Nacalai Tesque, Kyoto, Japan) was used for detection, according to the manufacturer’s protocol.

### Ultraviolet (UV) melting analysis

The melting temperature, *T*_m_ value, of each SSO was measured according to our previous work [12]. In brief, each SSO and native RNA oligonucleotide with complementary strands were dissolved in 10 mM sodium phosphate buffer (pH 7.2) containing 10 mM NaCl at 2 μM. The absorbance at 260 nm was measured from 5 °C to 95 °C at a scan rate of 0.5 °C/min. The peak temperature in the derivative curve is detected as the melting temperature (*T*_m_ value).

### Statistical analysis

Data are expressed as the mean ± standard deviation (SD). Statistical analyses were performed using EZR software [39]. The data were analyzed by ANOVA and, if this was statistically significant, group differences were analyzed using Dunnett’s multiple comparison test.

## Results

### Establishment of a tri-chromatic reporter cell line and SSO screening

**Fig 1:**
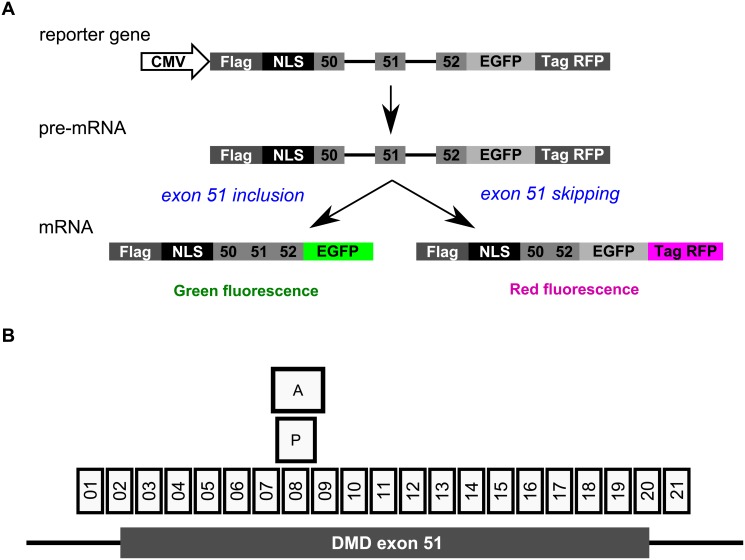
Schematic representation of the bi-chromatic reporter gene and SSOs targeting human *DMD* exon 51 used in the study. A) The bi-chromatic reporter gene encodes a Flag-tag, NLS, human dystrophin gene, two fluorescent proteins, and the *EGFP* and *TagRFP* genes. The EGFP-conjugated proteins are expressed when *DMD* exon 51 is included. Conversely, TagRFP-conjugated proteins are expressed when *DMD* exon 51 is skipped. B) The boxes represent the SSOs that were used in the assays. The boxes with numbers (01–21) indicate LNA-based SSOs, while the others represent AVI-4658 (A) and PRO-051 (P). Each SSO contains a sequence that is complementary to the human *DMD* gene.

To establish the tri-chromatic reporter cell line, we initially constructed a bi-chromatic reporter gene [20]. As shown in Fig 1A, the bi-chromatic reporter cell line encodes the *DMD* minigene conjugated with a Flag-tag, NLS, EGFP, and TagRFP. We used the combination of two fluorescent proteins, EGFP and TagRFP, for our reporter system because of the types of LED-based illumination limitations of ToxInsight. When *DMD* exon 51 (233 bp) is included after pre-mRNA splicing, a stop codon arises at the end of the *EGFP* gene, and EGFP-conjugated proteins are expressed. Conversely, when *DMD* exon 51 is skipped, a frame-shift triggers the loss of the stop codon at the end of the *EGFP* gene. Because a particular feature of the *EGFP* gene, namely that the −1 reading frame contains no stop codons, the *TagRFP* mRNA is translated and TagRFP rather than EGFP-conjugated proteins are expressed.

In the tri-chromatic reporter cell line, the stable expression of blue fluorescent proteins is used for the normalization of the data, by detecting the cell shape and counting the number of cells using HCS. Thus, at first, we established cells that express stable TagBFP proteins (Flp-In CHO-BFP cells) in Flp-In CHO cells by transfecting the pTagBFP-N vector and selecting cells that grow in the presence of G-418. Then, we established the tri-chromatic reporter cell line using Flp-In CHO-BFP cells and the Flp-In system in our bi-chromatic reporter plasmids.

**Fig 2:**
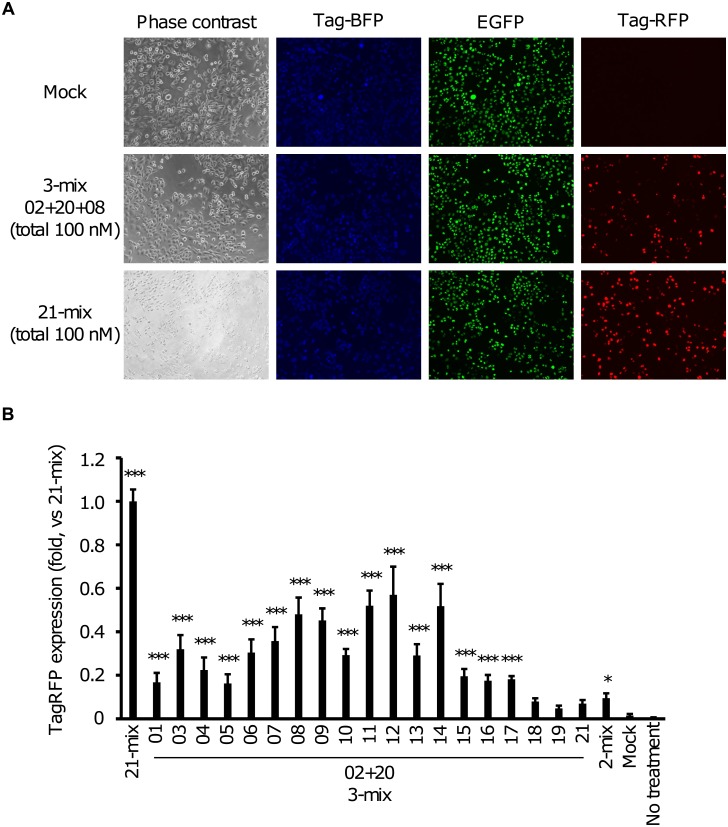
Screening of 3-mix LNA-based SSO cocktails using the established reporter cell line. A) Phase contrast and fluorescence microscopy images of SSO-transfected reporter cells; 21-mix SSOs (100 nM total), 3-mix (02+20+08) SSOs (100 nM total), and mock-transfected cells were analyzed using a BZ-8000 microscope. Fluorescence microscopy, showing that only SSO-transfected reporter cells display red fluorescence. Phase contrast: phase contrast images, TagBFP: blue fluorescence images using the (Ex/Em = 360/460 nm) filter, EGFP: green fluorescence images using the (Ex/Em = 470/525 nm) filter, and TagRFP: red fluorescence images using the (Ex/Em = 545/605 nm) filter. Mock: treated with Lipofectamine 2000 only. Each analysis was duplicated and repeated five times to ensure the reproducibility of the results. B) TagRFP expression levels, measured by HCS. The graph shows the normalized red fluorescence intensity, relative to the value in the 21-mix SSO-transfected cells (set at 1). Values represent the mean ± standard deviation of five independent experiments performed in duplicate. Data were analyzed by ANOVA (*P* < 0.001). Significant differences compared to the mock were determined using Dunnett’s test and are indicated by asterisks (*P < 0.05, ***P < 0.001). Mock: treated with Lipofectamine 2000 only; no treatment: no transfection.

We synthesized 21 LNA-based SSO sequences designed to tile across the entire target *DMD* exon 51 sequence for screening purposes (Fig 1B and S1 Table). All SSOs represent 13-mer LNA/DNA mixmers that contain six LNA analogs, as described in our previous report [12]. Next, we evaluated the efficacy of LNA-based SSOs in our established reporter cell line, by transfecting SSOs at a concentration of 100 nM. To determine exon skipping, we assessed the expression of fluorescent proteins. Unfortunately, we could not detect *DMD* exon 51 skipping events using single SSOs due to the low expression of red fluorescent proteins (S1 Fig). A previous study has reported that a cocktail of SSOs can modulate splicing more efficiently than single SSOs, but this depends on the targeting exon length and sequence [40]. To assess the efficacy of such a SSO cocktail in our system, we employed this strategy for SSO screening. At first, we investigated the exon skipping activity of the LNA-based SSO cocktail that contains equal amounts of all 21 SSOs (designated as the 21-mix). The 21-mix LNA-based SSO cocktail showed a markedly higher skipping activity (S1 Fig) and we could detect red fluorescence in cells transfected with the 21-mix LNA-based SSO cocktail (Fig 2A, 21-mix). On the other hand, we could not detect red fluorescence in mock-transfected reporter cells (Fig 2A, mock). Both blue and green fluorescence were detected in both cell types (Fig 2A, 21-mix and mock). Thus, we considered the 21-mix LNA-based SSO cocktail as the positive control in the following experiments.

Next, we evaluated the exon skipping activity of a mixture containing SSO 02 and 20 (designated as 2-mix), because we have previously shown that LNA-based SSOs targeting exon-intron junctions show a high skipping efficiency for *DMD* exon 58. The 2-mix LNA-based SSO cocktail showed a low exon skipping activity (S1 and S2 Figs). Therefore, we mixed one additional SSO in the 2-mix (designated as 3-mix) to explore possible SSO target sites and potentially enhance the exon skipping activity. The 3-mix LNA-based SSO cocktail containing equal amounts of the three LNA-based SSOs was transfected at a total concentration of 100 nM. As a result, many combinations of the 3-mix LNA-based SSO cocktails showed exon skipping activity (Fig 2B). Amongst them, the 3-mixes containing “02+20+08”, “02+20+09”, “02+20+11”, “02+20+12”, and “02+20+14” exhibited a higher skipping activity than other 3-mix LNA-based SSO cocktails. Conversely, the addition of SSOs targeting the 3′-region of exon 51 in *DMD* did not enhance the exon skipping activity when compared to that in the 2-mix LNA-based SSO cocktails. As shown in Fig 2A and S2 Fig, fluorescence microscopy revealed that reporter cells transfected with 3-mix LNA-based SSO cocktails express red fluorescent proteins. Thus, we validated that the reporter system could be used for the evaluation of SSOs.

### Characteristics of the established reporter cell line

We observed that the red fluorescence intensity in the established cell line was affected by the transfection of SSOs. Therefore, we sought to investigate whether the red fluorescence intensity originated from the conjugated fluorescent proteins as a result of changes in the splicing pattern of the reporter gene; to assess this, we employed western blotting, RT-PCR, quantitative RT-PCR, and HCS.

**Fig 3:**
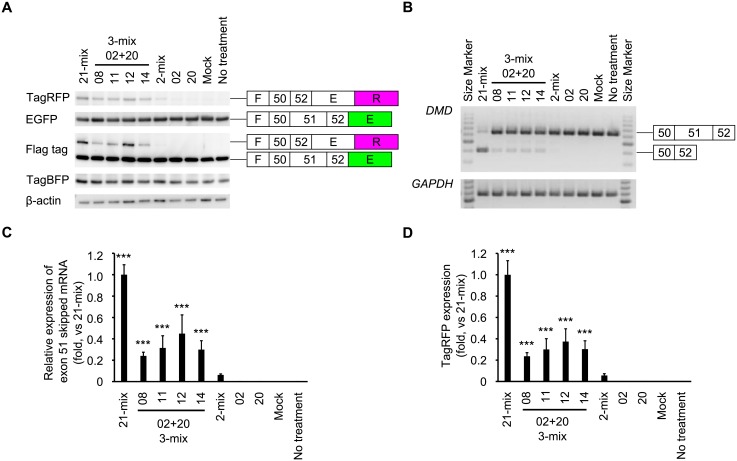
Characteristics of the established reporter cell line. The reporter cells were transfected with the indicated SSOs at 100 nM for 24 h. A) Expression levels of cellular Flag-tag-conjugated TagRFP (65.7 kDa), TagBFP (26.3 kDa), and β-actin proteins (40.3 kDa) analyzed by western blotting. Each analysis was duplicated and repeated three times to ensure the results were reproducible. B) RT-PCR analysis indicating an upper (637 bp) and a lower band (404 bp). *cGAPDH* was used as an internal control. Each analysis was duplicated and repeated three times to ensure the results were reproducible. C) Expression level of skipped *DMD* exon 51 mRNA, measured by quantitative RT-PCR. Values represent the mean ± standard deviation of three independent experiments performed in duplicate. Data were analyzed by ANOVA (*P* < 0.001). Significant differences compared to the mock were determined using Dunnett’s test and are indicated by asterisks (***P < 0.001). D) Levels of TagRFP expression measured by HCS. The normalized red fluorescence intensity, relative to the value in the 21-mix SSO-transfected cells was set as 1. Values represent the mean ± standard deviation of three independent experiments performed in duplicate. Data were analyzed by ANOVA (*P* < 0.001). Significant differences compared to the mock were determined using Dunnett’s test and are indicated by asterisks (***P < 0.001).

At first, we performed western blotting to detect Flag-tagged fluorescent proteins. Both the anti-EGFP and anti-Flag-tag antibodies can detect Flag-tag-conjugated EGFP proteins (46.6 kDa) that are translated when exon 51 is included in the reporter mRNA. The Flag-tag-conjugated EGFP proteins were detected in all tested cells (Fig 3A). Conversely, both the anti-TagRFP and anti-Flag-tag antibodies can detect Flag-tag-conjugated TagRFP proteins (65.2 kDa) that are translated from the reporter mRNA containing the skipped exon. Notably, the Flag-tagged red fluorescent proteins could only be detected in cells transfected with the 21-mix and 3-mix LNA-based SSO cocktails (Fig 3A). As internal controls, we also detected the expression of both TagBFP (26.3 kDa) and β-actin (41.7 kDa). As shown in S3 Fig, the histograms of the protein expression for both TagRFP and EGFP in reporter cells were obtained using HCS. In this analysis, we could confirm that the number of cells that express TagRFP proteins increased after treatment with LNA-based SSO cocktails. Thus, our western blot analyses are in agreement with the exon skipping activity detected in 3-mix LNA-based SSO cocktails using HCS.

Next, to investigate *DMD* exon 51 skipping, we performed RT-PCR (Fig 3B and S4 Fig). Exon-skipped bands were detected when each of the 3-mix LNA-based SSO cocktail was transfected. Moreover, the 21-mix LNA-based SSO cocktail showed the highest exon skipping activity. Thus, we confirmed that the fluorescent proteins were expressed in accordance with the splicing patterns of the reporter gene in the established reporter cell line, following the induction of exon skipping using SSOs.

To quantitatively assess SSO-induced exon skipping, quantitative RT-PCR was performed to detect the expression levels of exon 51-skipped mRNA (Fig 3C). The single LNA-based SSOs 02 and 20 did not display sufficient exon skipping activity. However, 2-mix LNA-based SSO cocktails induced exon skipping: the relative expression of exon 51-skipped mRNA, vs. the one detected in the 21-mix was 0.05. Furthermore, the 3-mix LNA-based SSO cocktails induced higher exon skipping activities: the relative expression of exon 51-skipped mRNA, vs. the one detected in the 21-mix was 0.24–0.33. The HCS results were also similar to those obtained using quantitative RT-PCR (Fig 3D). The differences in the expression levels of TagRFP proteins between cells transfected with each of the LNA-based SSO cocktails were successfully detected by HCS. Notably, the exon skipping activity of the 2-mix LNA-based SSO cocktail was still low but could be detected by HCS. Thus, the established reporter cell lines in combination with HCS allow for the quantitative assessment of SSO-induced exon skipping.

These results indicated that we can quickly detect SSO-induced exon skipping in living cells using this reporter system, without harvesting cells or performing multi-steps assays such as RT-PCR.

### Dose-dependent activity of 3-mix LNA-based SSO cocktails

**Fig 4:**
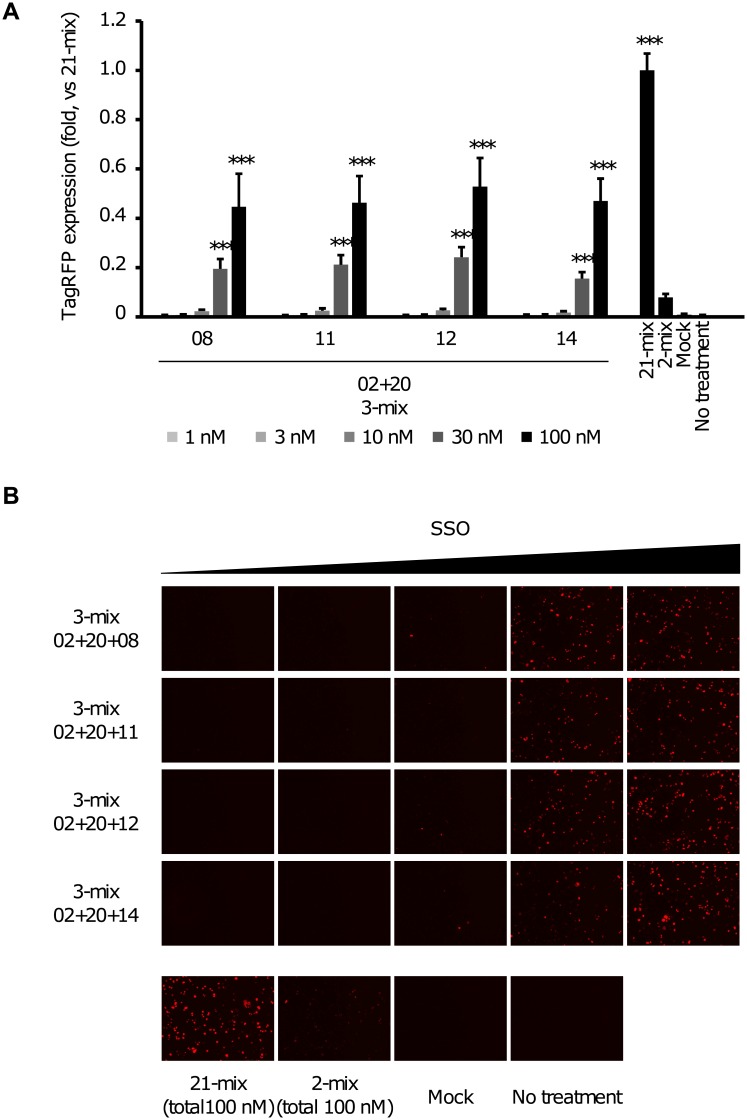
Dose-dependent activity of 3-mix LNA-based SSO cocktails. Reporter cells were transfected with the indicated concentrations of LNA-based SSO cocktails (1–100 nM) for 24 h. A) TagRFP expression, measured by HCS. The normalized red fluorescence intensity, relative to the value in the 21-mix SSO-transfected cells (set at 1). Values represent the mean ± standard deviation of four independent experiments performed in duplicate. Data were analyzed by ANOVA (*P* < 0.001). Significant differences compared to the mock were determined using Dunnett’s test and are indicated by asterisks (***P < 0.001). B) Fluorescence microscopy images of SSO-transfected reporter cells. The cells transfected with different concentrations of SSOs were analyzed using a BZ-8000 microscope. Fluorescence microscopy, demonstrating that the expression of TagRFP-conjugated proteins is dose-dependent. TagRFP: red fluorescence images using the (Ex/Em = 545/605 nm) filter. Mock: treated with Lipofectamine 2000 only. The analysis was duplicated and repeated four times to ensure the results were reproducible.

We next investigated the dose dependency of four LNA-based SSO cocktails: the “02+20+08”, “02+20+11”, “02+20+12” and “02+20+14” 3-mixes that showed higher skipping activities in our reporter system. The four LNA-based SSO cocktails were transfected into the reporter cell lines and the normalized intensity of the red fluorescence was evaluated by HCS. The final concentration ranged from 1 to 100 nM. Each LNA-based SSO cocktail induced exon skipping in a dose-dependent manner (Fig 4A). We also investigated the expression of the TagRFP-conjugated protein using fluorescence microscopy. As expected, the expression of red fluorescent proteins correlated with the dose of LNA-based SSOs cocktails (Fig 4B). Thus, this reporter system can also be used for investigating the dose-dependency of SSOs.

### Induction of exon 51 skipping in the endogenous human *DMD* transcript

**Fig 5:**
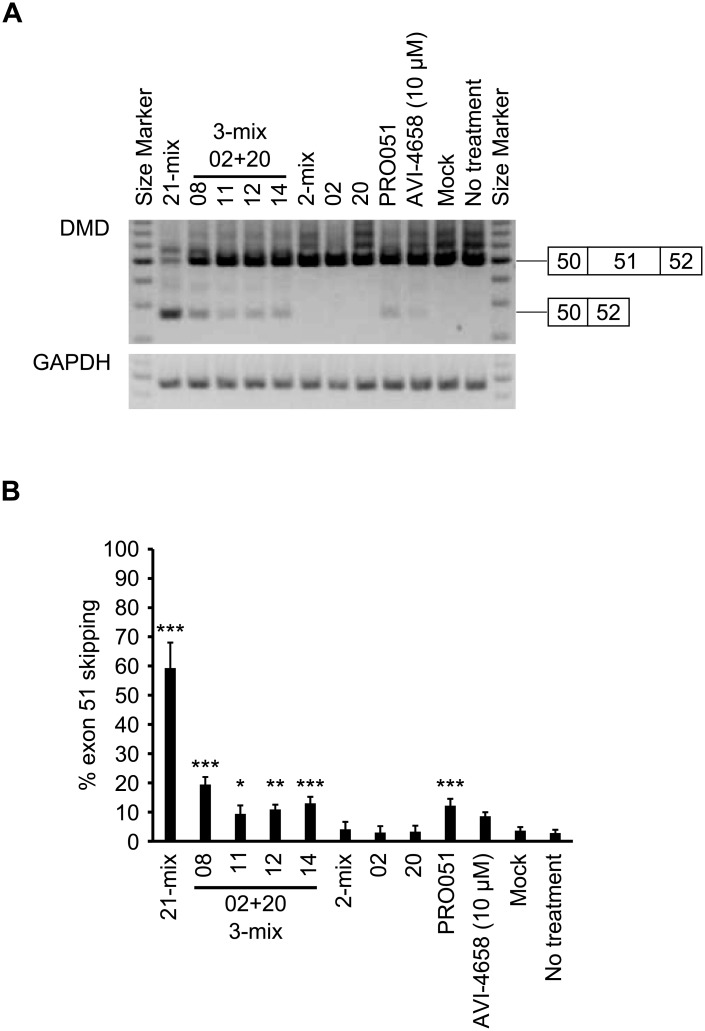
Induction of exon 51 skipping in the endogenous human *DMD* transcript. A) Differentiated RD cells were transfected with the indicated SSOs (500 nM). For AVI-4658 transfection, Endo-porter was used as the delivery reagent, and 10 μM of AVI-4658 was used. The RT-PCR analysis shows an upper (497 bp) and a lower band (264 bp). *GAPDH* was used as an internal control. The analysis was duplicated and repeated three times to ensure the results were reproducible. B) The % exon 51 skipping was calculated using the ImageJ software as the amount of exon-skipped transcript relative to the total amount of exon-skipped plus full-length transcripts. Values represent the mean ± standard deviation of three independent experiments performed in duplicate. Data were analyzed by ANOVA (*P* < 0.001). Significant differences compared to the mock were determined using Dunnett’s test and are indicated by asterisks (*P < 0.05, **P < 0.01, ***P < 0.001).

We next sought to evaluate the LNA-based SSO cocktails that showed higher exon skipping activity in our established reporter cell lines, in the context of the endogenous *DMD* mRNA. For this analysis, we used four 3-mix LNA-based SSO cocktails: “02+20+08”, “02+20+11”, “02+20+12” and “02+20+14”, which induce higher levels of exon skipping in a dose-dependent manner. We employed the human RD cell line, that can be differentiated by TPA stimulation [35, 36]. After 24 h following SSO transfection into the differentiated RD cells, the expression of *DMD* mRNA was measured by RT-PCR. Moreover, we evaluated the exon skipping activity of both PRO-051 and AVI-4658 (Fig 1B). PRO-051 is known as a 2′-*O*-methyl RNA-based SSO, while AVI-4658 is known as eteplirsen, a drug for patients with DMD, which was conditionally approved by the FDA in 2016 [3, 41, 42]. At first, we revealed that the 21-mix LNA-based SSO cocktail induced exon 51 skipping (the % exon skipping was 59.33%) (Fig 5A and 5B). We observed bands generated through exon skipping following treatment with the 3-mix LNA-based SSO cocktails (500 nM), and the exon skipping activity was as same as that observed following treatment with PRO-051 (500 nM) and AVI-4658 (10 μM) (Fig 5A and 5B).

Our data indicated that the established reporter cell lines represent an excellent assay system that enabled us to screen for SSO-induced exon skipping in the context of the endogenous *DMD* mRNA. Thus, this reporter systems can be a powerful tool for screening multiple SSOs.

## Discussion

This study aimed to develop a novel tri-chromatic reporter cell line for the evaluation of the exon skipping activity of LNA-based SSOs that could be applied for HCS. We have established a reporter cell line that expresses tri-chromatic fluorescent proteins for analyzing the *DMD* exon 51 skipping activity, and evaluated this screening system using various assays. We also performed a brief screening of LNA-based SSOs targeting *DMD* exon 51 and revealed that a cocktail of LNA-based SSOs shows a higher exon skipping activity.

First, we improved the existing reporter system for the evaluation of SSO-induced exon skipping activity using HCS. Recently, HCS has become a standard protocol for rapid screening assays [31–34]. In fact, HCS has often been used for exploring small molecules; however, to our knowledge, it is rarely used for the screening of SSOs. Using a bi-chromatic reporter system that has been previously described by other groups [20, 26–30], the exon skipping activity of SSOs was analyzed by taking the ratio of the expression of two fluorescent proteins which correlates with exon skipping. However, in this system it is impossible to normalize the exon skipping activity of SSOs by the number of cells. Nevertheless, to more precisely evaluate the exon skipping activity, a normalization approach that takes into account the cell number appears to be important because SSOs might induce aberrant splicing, which would be reflected by a lack of fluorescent proteins in a bi-chromatic reporter system. In our established tri-chromatic reporter cell line the TagBFP protein was stably expressed, and it enabled us to normalize the fluorescence intensity data by counting the number of cells using HCS (Fig 2A). Therefore, aberrant splicing will not affect our results. Furthermore, we also confirmed that reporter cells could be used for the screening of SSOs, as they express both EGFP- and TagRFP-conjugated *DMD* minigenes based on the splicing pattern of *DMD* exon 51 (Fig 1A).

Second, our data revealed that using our reporter cell line in combination with HCS led to similar results to those obtained using conventional methods for the evaluation of SSO-induced exon skipping, such as RT-PCR, quantitative RT-PCR, and western blotting (Fig 2). Additionally, this novel reporter cell line enables us to perform cost and time-effective SSO screens, because it is sufficient to detect the expression of fluorescent proteins by HCS; hence, we can evaluate exon skipping in living cells. Conversely, when using conventional methods such as RT-PCR, quantitative RT-PCR, and western blotting, it is necessary to pass through multiple steps like cell harvesting, cell homogenization, and gel loading. Therefore, our novel reporter cell line could be used as a rapid screening system and an alternative for current protocols. In this study, we demonstrate that the LNA-based SSO cocktails which were screened in our tri-chromatic reporter cell lines showed high exon skipping activities on endogenous *DMD* mRNA in RD cells. Similar to our results in tri-chromatic reporter cells, RT-PCR analyses in RD cells showed that the 3-mix cocktails induced a higher level of exon skipping than that induced by 2-mix cocktails and single SSOs (Figs 3 and 5). Thus, our tri-chromatic reporter cells could be used as an indicator of potential effects in the normal genomic context. To obtain SSOs with higher exon skipping activities, it is necessary to optimize the design of SSOs, such as target sites, GC content, melting temperature (*T*_m_ value), chemistry, and lengths, by screening many candidate SSOs. Our tri-chromatic reporter cells represent a versatile tool for the drug screening of splicing modulators in future studies.

Third, we performed the screening of a newly designed series of 13-mer LNA-based SSOs for *DMD* exon 51 skipping. In our previous work, we evaluated various LNA-based SSO lengths (seven to 23-mers) and revealed that a 13-mer LNA-based SSO could more efficiently induce *DMD* exon 58 skipping [12]. However, 13-mer LNA-based SSOs for *DMD* exon 51 skipping did not show sufficient exon skipping activity in this study (S1 Fig). One of the possible reasons that would account for this discrepancy is the different lengths of the targeted exons: *DMD* exon 51 is 233 bp long and *DMD* exon 58 is 121 bp long. Thus, one short SSO might prove insufficient for inhibiting the binding of splicing factors to *DMD* exon 51. According to previous work by *Wilton et al*., *DMD* exons were listed based on the difficulty of inducing exon skipping using 2′-OMe RNA-based SSOs, where 2′-OMe RNA-based 25-mer SSOs induced *DMD* exon 51 skipping [43]. Moreover, *Echigoya et al*. have recently reported that 30-mer PMO-based SSOs display an increased skipping of *DMD* exon 51 [44]. Conversely, we showed the potential for using a cocktail of 13-mer LNA-based SSOs. *Adams et al*. have previously reported that SSO cocktails showed higher exon skipping activity than single SSOs [40], by using 2′-*O*-methyl RNA-based or PMO-based SSOs for their cocktails. This study is, to our knowledge, the first to apply this strategy for LNA-based SSOs. Even though individual LNA-based SSOs did not effectively induce exon 51 skipping (S1 Fig), a mixture containing three LNA-based SSOs resulted in an increase in exon skipping activity (Fig 2A). When screening with 3-mix LNA-based SSO cocktails, the potential target sites for LNA-based SSOs were predicted with the ESE finder 3.0 (S5 Fig). Specifically, the target sites for 3-mix cocktails, including exon-intron junctions and internal sequences of the *DMD* exon 51, were predicted as high scores by the ESE finder 3.0. Thus, based on the targeted exon 51, we demonstrated that LNA-based SSO cocktails increase exon skipping.

For the future development of SSO based therapeutics, it is important to assess exon skipping activities *in vivo*. Although the *in vivo* exon skipping activity of LNA-based SSO cocktails has not yet been investigated, *Aoki* et al. have previously reported that PMO-based SSO cocktails showed high exon skipping activities in not only human primary fibroblast but also *mdx*52 mice [45]. Therefore, we might anticipate that LNA-based SSO cocktails also induce exon skipping efficiency *in vivo*. On the other hand, in order to apply LNA-based SSO cocktails in future therapeutic applications, it is important to assess the toxicity of SSOs. Specifically, it is crucial to consider potential off-target effects, as those observed when using other oligonucleotide-based therapeutics, such as siRNAs, miRNAs, and antisense oligonucleotides. Using *in silico* analyses, we confirmed that some LNA-based SSOs used in this study may target mRNAs that contain SSO-complementary sequences sites (S5 Table). To prevent the off-target effects of LNA-based SSOs, it will be necessary to optimize the target sites of each LNA-based SSO from an efficacy and toxicity viewpoint.

In conclusion, we established a novel tri-chromatic reporter cell line that could be used to evaluate SSO-induced exon skipping in addition to conventional methods. We also screened LNA-based SSO cocktails and revealed that they induce a high level of exon skipping at the endogenous *DMD* gene locus. Taken together, our data suggest that it is better to perform large-scale screens with many candidate SSOs, similar to those reported in our previous work [12]. To perform such large-scale screens, a rapid evaluation can be easily achieved using the tri-chromatic reporter cell line reported in this study.

## Supporting information

S1 Fig Screening of single SSOs using the reporter cell line. The reporter cells were transfected with the indicated SSOs at 100 nM, and the intensity of the TagRFP fluorescence in the reporter cells was measured using HCS analysis. The graph shows the normalized TagRFP fluorescence intensity, relative to the value in the 21-mix SSO-transfected cells (set at 1). Values represent the mean ± standard deviation of five independent experiments performed in duplicate. Mock: treated with Lipofectamine 2000 only; no treatment: no transfection.
(PDF)

S2 Fig The expression of EGFP- and TagRFP-conjugated proteins and TagBFP proteins in the reporter cell line. The indicated SSO-transfected (100 nM total) cells were analyzed using a BZ-8000 microscope. Phase contrast and fluorescence microscopy images of SSO-transfected reporter cells. TagRFP-conjugated proteins can only be detected in SSO-transfected cells. EGFP-conjugated proteins and TagBFP proteins can be detected in all cells. Phase contrast: phase contrast images, TagBFP: blue fluorescence images using the (Ex/Em = 360/460 nm) filter, EGFP: green fluorescence images using the (Ex/Em = 470/525 nm) filter, and TagRFP: red fluorescence images using the (Ex/Em = 545/605 nm) filter. Mock: treated with Lipofectamine 2000 only. The analysis was duplicated and repeated five times to ensure the results were reproducible.
(PDF)

S3 Fig Scatter-plot of the reporter cells after SSO transfection by HCS. Reporter cells seeded on 96-well black plates were transfected with the indicated SSOs at 100 nM. Twenty-four hours after transfection, fluorescence images of the reporter cell line were acquired using ToxInsight. The captured fluorescence images were analyzed using the Thermo Scientific Cellomics Spot Detector V4 program, to obtain scatter plots of all single cells in each well. The X axis shows the total intensity of EGFP-conjugated proteins in each cell, and the Y axis shows the total intensity of TagRFP-conjugated proteins in each cell. The analysis was duplicated and repeated five times to ensure the results were reproducible.
(PDF)

S4 Fig Exon skipping activity of 3-mix LNA-based SSO cocktails using the established reporter cell line. Reporter cells were transfected with the indicated SSOs at 100 nM and incubated for 24 h. The % exon 51 skipping was calculated as the amount of exon skipped transcript relative to the total amount of exon skipped plus full-length transcripts. Values represent the mean ± standard deviation of three independent experiments performed in duplicate.
(PDF)

S5 Fig Estimation of splice factor binding sites in the human *DMD* exon 51. Potential exonic splicing enhancer (ESE) sites of splice factors SRSF1, SRSF1 (IgM-BRCA1), SRSF2, SRSF5, and SRSF6 in human *DMD* exon 51 (including 50 bp of the flanking intronic sequence). These ESE sites are predicted by ESE finder 3.0 [46]. The predicted ESE sequences are candidate SSO target sites for inducing exon skipping.
(PDF)

S1 Table SSOs used for the assay. Twenty-one LNA-based SSOs, PRO-051, and AVI-4658 for dystrophin exon 51 skipping are shown. Sequences are shown from 5′ to 3′. Capital letters with (L); LNA. Small letters: DNA. Capital letters with (M); 2′-OMe RNA. Capital letters with (P); PMO. ^; phosphorothioate backbone. For *T*_m_ analysis, SSOs and complementary native RNAs were diluted in 10 mM phosphate buffer (pH 7.2), 10 mM NaCl at 2 μM. Values represent the mean ± standard deviation of three independent experiments.
(PDF)

S2 Table Primers used for PCR analysis for tri-chromatic reporter cell line. Sequences for the forward (For.) and reverse (Rev.) primers for each target are shown. Sequences are shown from 5′ to 3′.
(PDF)

S3 Table Primers used for PCR analysis for RD cell line. Sequences for the forward (For.) and reverse (Rev.) primers for each target are shown. Sequences are shown from 5′ to 3′.
(PDF)

S4 Table Primers used for quantitative PCR analysis for reporter cell line. Sequences for the forward (For.) and reverse (Rev.) primers for each target are shown. Sequences are shown from 5′ to 3′.
(PDF)

S5 Table A number of transcripts that contain sequence complementary to SSOs. To investigate the number of transcripts that contain a fully matched sequence to the target SSO sequences, we used GGRNA, a Google-like fast search engine for genes and transcripts (http://GGRNA.dbcls.jp/) [47]. In this analysis, splicing variants with the same gene ID were considered as one gene and the number of transcripts of the DMD gene was excluded.
(PDF)
