# Supplementary figures and images for: Construction of a tri-chromatic reporter cell line for the rapid and simple screening of splice-switching oligonucleotides targeting DMD exon 51 using high content screening
Source: PLoS One. 2018 May 16;13(5):e0197373. doi: 10.1371/journal.pone.0197373 (PMC5955590; doi:10.1371/journal.pone.0197373)

S1 Fig

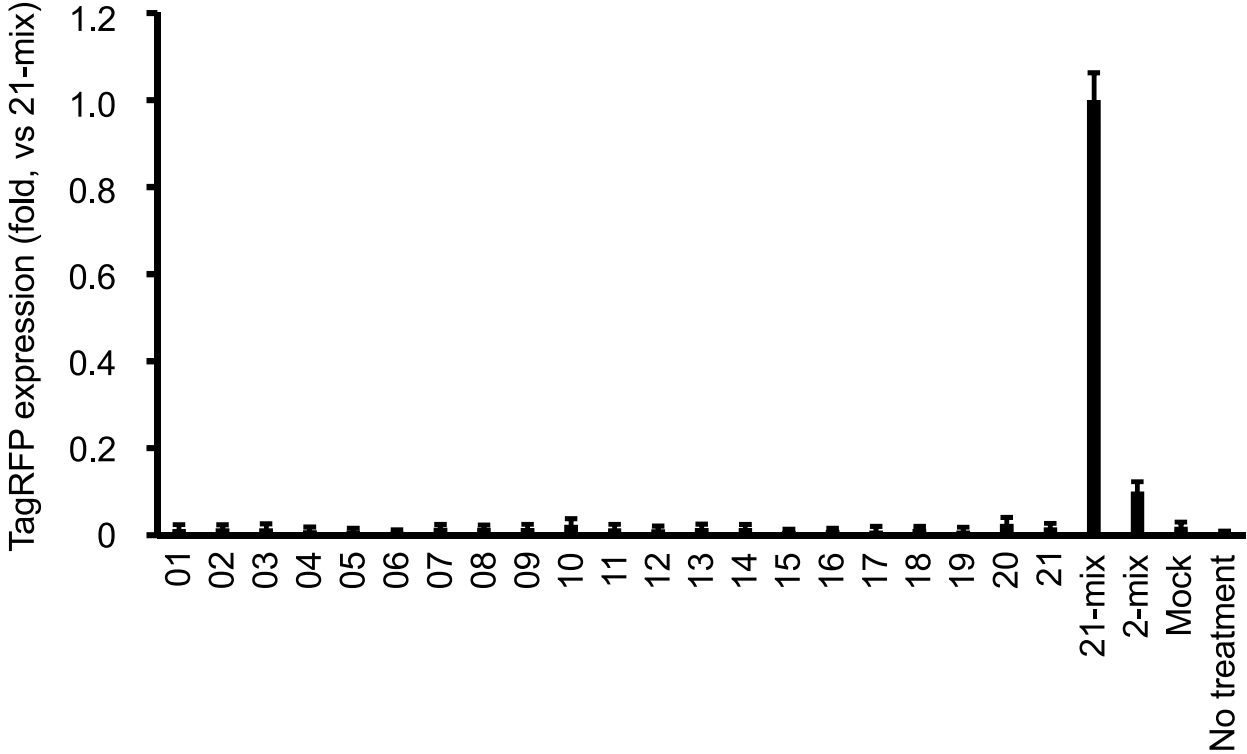

Supplement: S1 Fig — The reporter cells were transfected with the indicated SSOs at 100 nM, and the intensity of the TagRFP fluorescence in the reporter cells was measured using HCS analysis. The graph shows the normalized TagRFP fluorescence intensity, relative to the value in the 21-mix SSO-transfected cells (set at 1). Values represent the mean ± standard deviation of five independent experiments performed in duplicate. Mock: treated with Lipofectamine 2000 only; no treatment: no transfection. (PDF) [file pone.0197373.s001.pdf]

S2 Fig

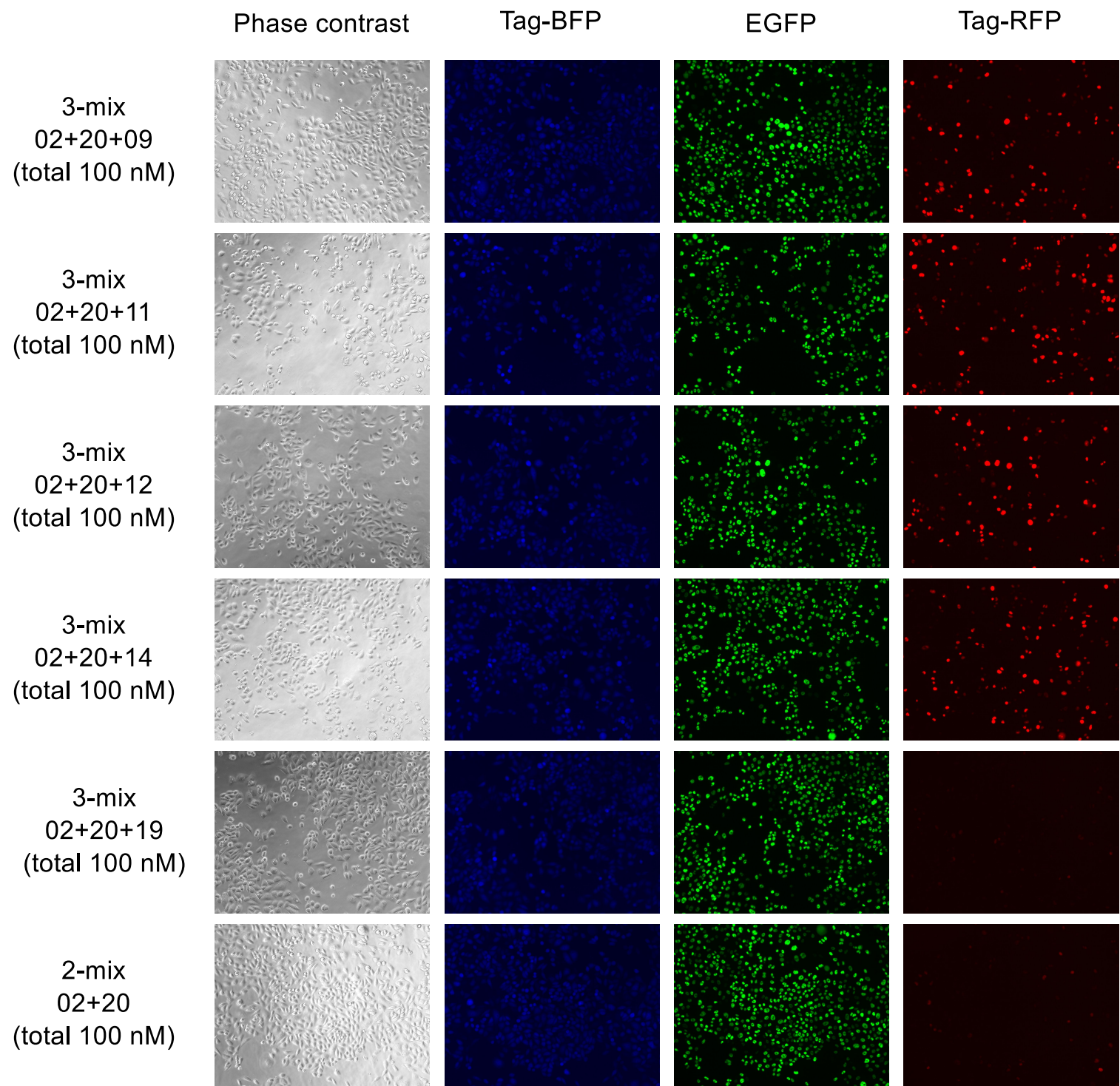

Supplement: S2 Fig — The indicated SSO-transfected (100 nM total) cells were analyzed using a BZ-8000 microscope. Phase contrast and fluorescence microscopy images of SSO-transfected reporter cells. TagRFP-conjugated proteins can only be detected in SSO-transfected cells. EGFP-conjugated proteins and TagBFP proteins can be detected in all cells. Phase contrast: phase contrast images, TagBFP: blue fluorescence images using the (Ex/Em = 360/460 nm) filter, EGFP: green fluorescence images using the (Ex/Em = 470/525 nm) filter, and TagRFP: red fluorescence images using the (Ex/Em = 545/605 nm) filter. Mock: treated with Lipofectamine 2000 only. The analysis was duplicated and repeated five times to ensure the results were reproducible. (PDF) [file pone.0197373.s002.pdf]

S3 Fig

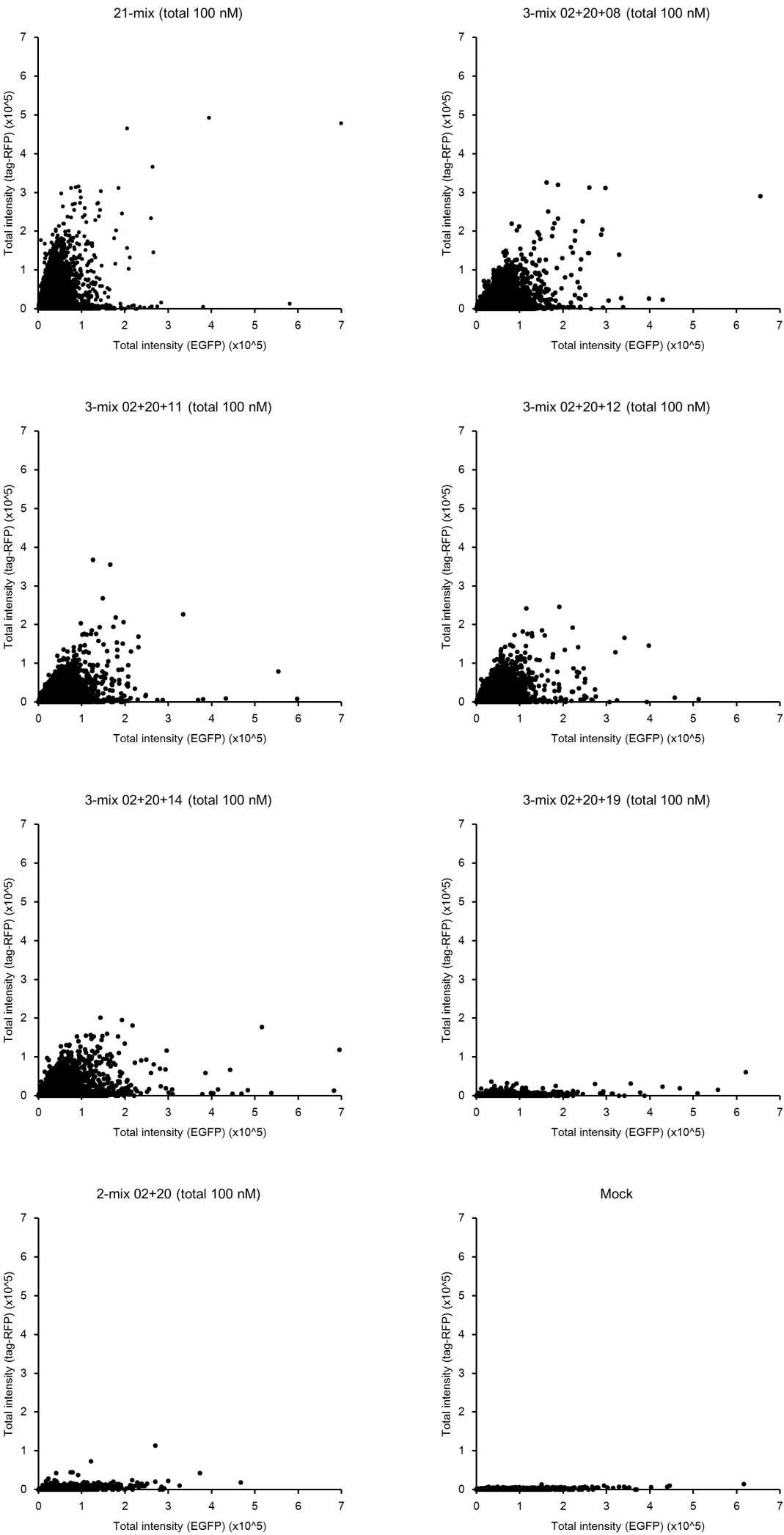

Supplement: S3 Fig — Reporter cells seeded on 96-well black plates were transfected with the indicated SSOs at 100 nM. Twenty-four hours after transfection, fluorescence images of the reporter cell line were acquired using ToxInsight. The captured fluorescence images were analyzed using the Thermo Scientific Cellomics Spot Detector V4 program, to obtain scatter plots of all single cells in each well. The X axis shows the total intensity of EGFP-conjugated proteins in each cell, and the Y axis shows the total intensity of TagRFP-conjugated proteins in each cell. The analysis was duplicated and repeated five times to ensure the results were reproducible. (PDF) [file pone.0197373.s003.pdf]

S4 Fig

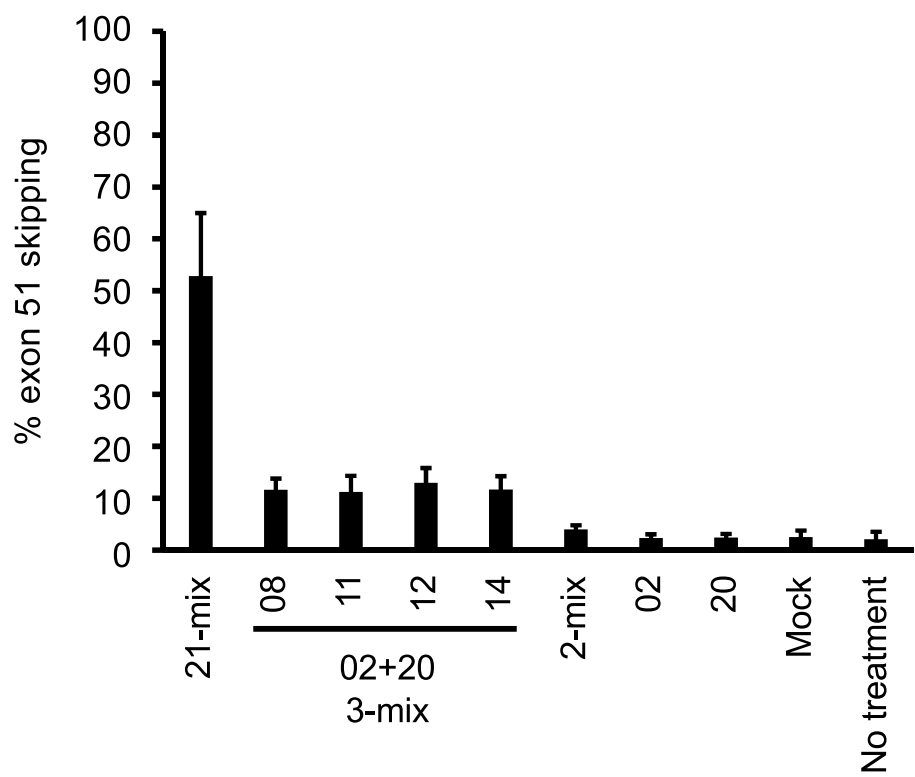

Supplement: S4 Fig — Reporter cells were transfected with the indicated SSOs at 100 nM and incubated for 24 h. The % exon 51 skipping was calculated as the amount of exon skipped transcript relative to the total amount of exon skipped plus full-length transcripts. Values represent the mean ± standard deviation of three independent experiments performed in duplicate. (PDF) [file pone.0197373.s004.pdf]

S5 Fig

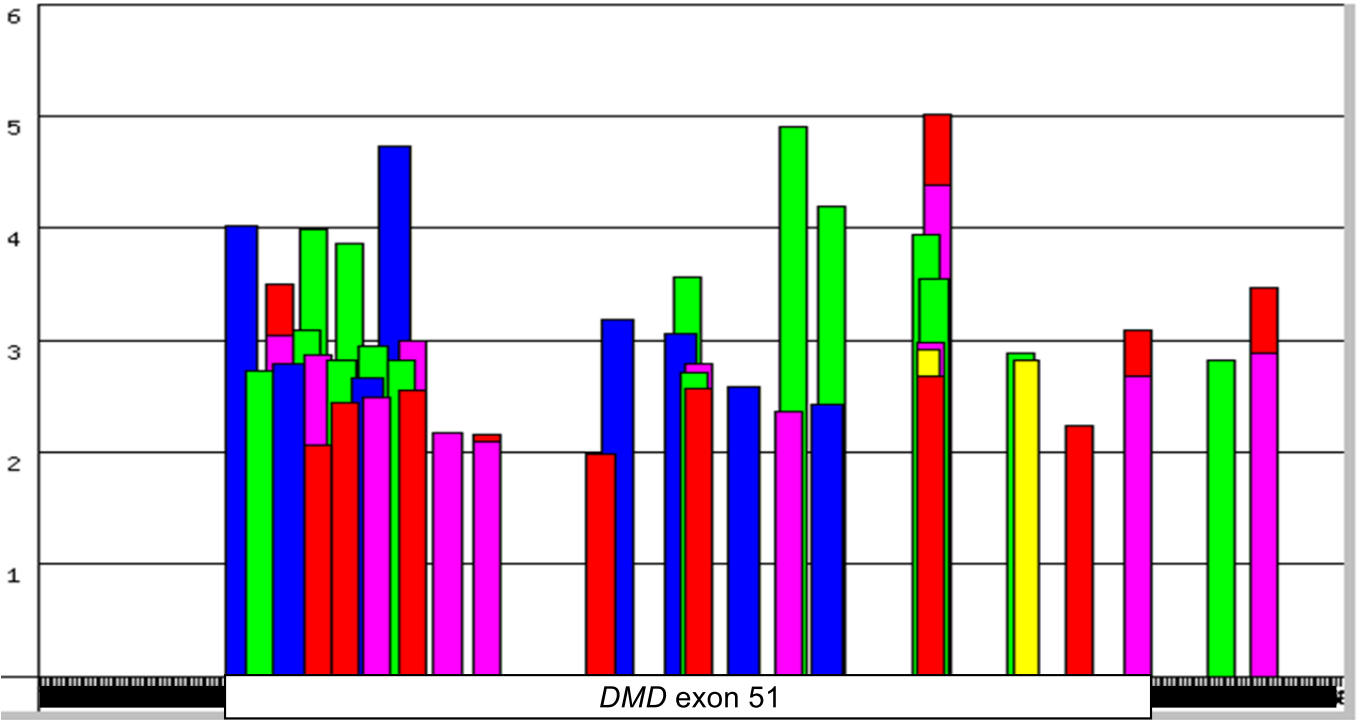

- SRSF1
- SRSF1 (IgM-BRCA1)
- SRSF2
- SRSF5
- SRSF6

Supplement: S5 Fig — Potential exonic splicing enhancer (ESE) sites of splice factors SRSF1, SRSF1 (IgM-BRCA1), SRSF2, SRSF5, and SRSF6 in human DMD exon 51 (including 50 bp of the flanking intronic sequence). These ESE sites are predicted by ESE finder 3.0 [46]. The predicted ESE sequences are candidate SSO target sites for inducing exon skipping. (PDF) [file pone.0197373.s005.pdf]
