# Supplementary material for: Construction of a tri-chromatic reporter cell line for the rapid and simple screening of splice-switching oligonucleotides targeting DMD exon 51 using high content screening
Source: PLoS One. 2018 May 16;13(5):e0197373. doi: 10.1371/journal.pone.0197373 (PMC5955590; doi:10.1371/journal.pone.0197373)
Supplement: S1 Table — Twenty-one LNA-based SSOs, PRO-051, and AVI-4658 for dystrophin exon 51 skipping are shown. Sequences are shown from 5′ to 3′. Capital letters with (L); LNA. Small letters: DNA. Capital letters with (M); 2′-OMe RNA. Capital letters with (P); PMO. ^; phosphorothioate backbone. For Tm analysis, SSOs and complementary native RNAs were diluted in 10 mM phosphate buffer (pH 7.2), 10 mM NaCl at 2 μM. Values represent the mean ± standard deviation of three independent experiments. (PDF) [file pone.0197373.s006.pdf]

S1 Table. SSOs used for the assay.

Twenty-one LNA-based SSOs, PRO-051, and AVI-4658 for dystrophin exon 51 skipping are shown. Sequences are shown from 5′ to 3′. Capital letters with (L); LNA. Small letters: DNA. Capital letters with (M); 2′-OMe RNA. Capital letters with (P); PMO. ^; phosphorothioate backbone. For T_m analysis, SSOs and complementary native RNAs were diluted in 10 mM phosphate buffer (pH 7.2), 10 mM NaCl at 2 μM. Values represent the mean ± standard deviation of three independent experiments.

Entry	Target	Sequence	T_m value (°C)
01	DMD exon51 -20-8	a^T(L)^t^T(L)^t^G(L)^g^G(L)^t^T(L)^t^T(L)^t	55.1 ± 0.3
02	DMD exon51 -7+6	t^A(L)^g^G(L)^a^G(L)^c^T(L)^a^A(L)^a^A(L)^t	54.0 ± 0.7
03	DMD exon51 +7+19	g^T(L)^a^A(L)^c^A(L)^g^T(L)^c^T(L)^g^A(L)^g	64.6 ± 0.3
04	DMD exon51 +20+32	t^T(L)^g^T(L)^g^T(L)^c^A(L)^c^C(L)^a^G(L)^a	70.0 ± 0.3
05	DMD exon51 +33+45	t^A(L)^g^T(L)^a^A(L)^c^C(L)^a^C(L)^a^G(L)^g	66.7 ± 0.2
06	DMD exon51 +46+58	a^T(L)^g^G(L)^c^A(L)^g^T(L)^t^T(L)^c^C(L)^t	70.3 ± 0.4
07	DMD exon51 +59+71	t^T(L)^c^T(L)^a^G(L)^t^T(L)^t^G(L)^g^A(L)^g	62.3 ± 0.7
08	DMD exon51 +72+84	a^G(L)^g^A(L)^a^G(L)^a^T(L)^g^G(L)^c^A(L)^t	67.7 ± 0.4
09	DMD exon51 +85+97	a^C(L)^c^T(L)^c^C(L)^a^A(L)^c^A(L)^t^C(L)^a	68.5 ± 0.3
10	DMD exon51 +98+110	t^G(L)^c^C(L)^a^G(L)^a^G(L)^c^A(L)^g^G(L)^t	77.9 ± 0.5
11	DMD exon51 +111+123	c^C(L)^c^G(L)^g^T(L)^t^G(L)^a^A(L)^a^T(L)^c	66.1 ± 0.5
12	DMD exon51 +124+136	a^G(L)^t^T(L)^c^T(L)^g^T(L)^c^C(L)^a^A(L)^g	69.4 ± 0.8
13	DMD exon51 +137+149	a^A(L)^g^C(L)^c^A(L)^g^T(L)^c^G(L)^g^T(L)^a	74.8 ± 0.4

Entry	Target	Sequence	T_m value (°C)
14	DMD exon51 +150+162	g^A(L)^t^C(L)^a^A(L)^g^C(L)^a^G(L)^a^G(L)^a	64.1 ± 0.6
15	DMD exon51 +163+175	g^A(L)^t^T(L)^t^T(L)^a^T(L)^a^A(L)^c^T(L)^t	42.7 ± 0.9
16	DMD exon51 +176+188	c^A(L)^t^C(L)^a^C(L)^c^C(L)^t^C(L)^t^G(L)^t	74.2 ± 0.8
17	DMD exon51 +189+201	c^A(L)^a^G(L)^g^T(L)^c^A(L)^c^C(L)^c^A(L)^c	73.7 ± 0.5
18	DMD exon51 +202+214	t^C(L)^g^T(L)^t^G(L)^a^T(L)^a^T(L)^c^C(L)^t	62.9 ± 0.5
19	DMD exon51 +215+227	c^T(L)^t^G(L)^a^T(L)^g^A(L)^t^C(L)^a^T(L)^c	55.0 ± 0.4
20	DMD exon51 +228-7	c^T(L)^c^A(L)^t^A(L)^c^C(L)^t^T(L)^c^T(L)^g	64.1 ± 0.2
21	DMD exon51 -8-20	t^T(L)^t^A(L)^t^C(L)^a^T(L)^t^T(L)^t^T(L)^t	41.7 ± 0.7
22	PRO-051	U(M)^C(M)^A(M)^A(M)^G(M)^G(M)^A(M)^A(M)^G(M)^A(M)^U(M)^G(M)^G(M)^C(M)^A(M)^U(M)^U(M)^U(M)^C(M)^U(M)	-
23	AVI-4658	C(P)T(P)C(P)C(P)A(P)A(P)C(P)A(P)T(P)C(P)A(P)A(P)G(P)G(P)A(P)A(P)G(P)A(P)T(P)G(P)G(P)C(P)A(P)T(P)T(P)T(P)C(P)T(P)A(P)G(P)	-
