# Supplementary material for: Construction of a tri-chromatic reporter cell line for the rapid and simple screening of splice-switching oligonucleotides targeting DMD exon 51 using high content screening
Source: PLoS One. 2018 May 16;13(5):e0197373. doi: 10.1371/journal.pone.0197373 (PMC5955590; doi:10.1371/journal.pone.0197373)
Supplement: S2 Table — Sequences for the forward (For.) and reverse (Rev.) primers for each target are shown. Sequences are shown from 5′ to 3′. (PDF) [file pone.0197373.s007.pdf]

S2 Table. Primers used for PCR analysis for tri-chromatic reporter cell line

Sequences for the forward (For.) and reverse (Rev.) primers for each target are shown. Sequences are shown from 5′ to 3′.

Gene	ID	Sequence	Size
DMD mini gene	For. primer	ACTTCAAGAGCTGAGGGCAA	637 bp (exon 51 inclusion)
	Rev. primer	CTTCATGTGGTCGGGGTAGC	404 bp (exon 51 skipped)
cGAPDH	For. primer	GGAAGGACTCATGACCACGG	584 bp
	Rev. primer	TGGGGGTTATTGGACAGGGA	
