# Supplementary material for: Construction of a tri-chromatic reporter cell line for the rapid and simple screening of splice-switching oligonucleotides targeting DMD exon 51 using high content screening
Source: PLoS One. 2018 May 16;13(5):e0197373. doi: 10.1371/journal.pone.0197373 (PMC5955590; doi:10.1371/journal.pone.0197373)
Supplement: S3 Table — Sequences for the forward (For.) and reverse (Rev.) primers for each target are shown. Sequences are shown from 5′ to 3′. (PDF) [file pone.0197373.s008.pdf]

**S3 Table. Primers used for PCR analysis for RD cell line.**

Sequences for the forward (For.) and reverse (Rev.) primers for each target are shown. Sequences are shown from 5′ to 3′.

| Gene | ID | Sequence | Size |
|---|---|---|---|
| *hDMD* 1st PCR | For. primer | GGACCATTTGACGTTAAGGAAAC | |
| | Rev. primer | AGGTCTTTGGCCAACTGCTTG | |
| *hDMD* 2nd PCR | For. primer | CACTCAGCCAGTGAAGAGGA | 497 bp (exon 51 inclusion) |
| | Rev. primer | ACTGATTCTGAATTCTTTCAATTCG | 264 bp (exon 51 skipped) |
| *hGAPDH* | For. primer | ACCACAGTCCATGCCATCAC | 452 bp |
| | Rev. primer | TCCACCACCCTGTTGCTGTA | |
