# Supplementary material for: Construction of a tri-chromatic reporter cell line for the rapid and simple screening of splice-switching oligonucleotides targeting DMD exon 51 using high content screening
Source: PLoS One. 2018 May 16;13(5):e0197373. doi: 10.1371/journal.pone.0197373 (PMC5955590; doi:10.1371/journal.pone.0197373)
Supplement: S5 Table — To investigate the number of transcripts that contain a fully matched sequence to the target SSO sequences, we used GGRNA, a Google-like fast search engine for genes and transcripts (http://GGRNA.dbcls.jp/) [47]. In this analysis, splicing variants with the same gene ID were considered as one gene and the number of transcripts of the DMD gene was excluded. (PDF) [file pone.0197373.s010.pdf]

S5 Table. A number of transcripts that contain sequence complementary to SSOs.

To investigate the number of transcripts that contain a fully matched sequence to the target SSO sequences, we used GGRNA, a Google-like fast search engine for genes and transcripts (http://GGRNA.dbcls.jp/) [47]. In this analysis, splicing variants with the same gene ID were considered as one gene and the number of transcripts of the DMD gene was excluded.

Entry	ID	Length of SSO	No. of transcripts containing the target sequence
02	DMD exon51-7+6	13	3
08	DMD exon51+72+84	13	8
11	DMD exon51+111+123	13	0
12	DMD exon51+124+136	13	4
14	DMD exon51+150+162	13	0
20	DMD exon51+228-7	13	2
22	PRO-051	20	0
23	AVI-4658	30	0
